# The Water Supply of Augusta

**Published:** 1877-06

**Authors:** Wm. H. Doughty

**Affiliations:** Augusta, Ga.


ATLANTA

MEDICAL AND SURGICAL JOURNAL

VOL. XV.] JUNE—1877. [No. 3

### Original Communications.

### THE WATER SUPPLY OF AUGUSTA: THE NECESSITY FOR ITS PROTECTION AGAINST FURTHER AND FUTURE CONTAMINATION.

BEING THE REPORT OF THE "SECTION ON SANITARY POLICE," READ AT THE REGULAR SESSION OF THE AUGUSTA LIBRARY AND MEDICAL SOCIETY HELD AT AUGUSTA, GA, APRIL 27, 1877.

BY WM. H. DOUGHTY, M.D.

*Mr. President:*

From the title of the section of which this is the first report, namely, "Sanitary *Police*," I shall assume that it was the design of the Society to elicit such action on the part of the reporter as would present the sanitary defects and wants of this municipality, rather than a mere rehearsal of current topics in general hygiene. The practical results to which such a line of duty leads are obvious; for, aside from the interest and pride naturally felt by the profession in the illustration and development of home problems in sanitary science, there is the great end which all should covet, namely, the enlightenment and commitment, if possible, of the public mind to the necessity for and value of sanitary reforms. That it is the desire of this Society that its deliberations on this and all kindred subjects shall be productive of this great good, I shall not question. The subjects pertaining to this section are those concerning which the public intelligence should be thoroughly quickened, since it is the only department of the science within the grasp of the unprofessional community, who, however willing to brave the risks of even the most empirical medication in person, too often prove impervious to all appeals for the correction of evils that prejudice the public health. For *preventive* medicine they entertain a degree of skepticism which accords it the fate of a refined subtlety, and consigns it to the care of theorists, whose suggestions are worthy only of a respectful hearing—rarely of adoption.

Experience shows that in this sphere the authority of the profession is almost a nullity, without the active interest of the civil authorities who represent the people in power. The necessity of the case renders it, therefore, neither unwise nor unprofessional to discuss even before them and for their benefit the problems at interest. So great is the liberty, or rather the license, of the people in this democratic government of ours, they may shut their ears even against professional remonstrance: that they need instruction will be admitted readily. I trust, therefore, that you will not deem it beneath the dignity of this body, nor ascribe it to any mean ambition on my part, if I shall hope ultimately to effect, in part at least, to the honor of this Society, a wholesome revulsion in the public mind upon this subject. Certainly it is a legitimate work, a laudable undertaking. Allow me to say, that if you would obtain recognition and acquire influence as a society with your fellow-citizens, it is at your command, by showing a capacity for promoting reform by useful suggestions, presented in such a manner as to create a public sentiment strongly in favor of their adoption as, perhaps, vital necessities. Efforts at administrative reform are powerless unless approved, if not incited, by a strong public sentiment; and again, executive indifference is powerless to resist a torrent of public feeling forcibly directed against it. Be it our task, then, first to create, if possible, this public sentiment, and then to direct it so forcibly against the abuses that exist as to exact from those in power the reform so greatly needed in the sanitary condition of the city.

In England, sanitary science has the enduring basis of organic law, which provides not simply for a Bureau of Health and Health Minister, so to speak, to enforce its authority upon individuals, but also to compel the performance of sanitary duty by corporate authorities. English enactments are not merely advisory, but compulsory in their operation. By the

"Artisan's and Laborer's Dwelling Act"* of 1875, according to Dr. Fulsom, the "local authorities are not only allowed to tear down buildings unfit for use as dwellings, and to sell the land if the owner refuses to place proper buildings thereon, but, under certain circumstances, they can be compelled to do so by the central authority."

The present I believe to be an opportune moment for pressing the consideration of necessary reforms, as the public mind is predisposed to entertain them; and to their credit be it spoken, the civil authorities have already taken a step forward by a re-organization of the Board of Health. I shall endeavor, in the discharge of my duty, in presenting serially the several important subjects connected with the local administration of the laws of hygiene, to keep in view such considerations as will lead to recommendations of practical value by this Society to the Board of Health and civil authorities.

The subject to which I invite attention this evening is, the "Water Supply of Augusta; the Necessity for its Protection against further and future Contamination."

The study of the geography of disease indicates conclusively the great fact, that the proportion of diseases and deaths due to climatic causes is largely exceeded by those of artificial, or social, origin—a significant comment, and, at the same time, a terrible satire, upon what is complacently called the *civilization* of mankind. This fact, generally admitted, propounds, for solution, the most difficult problem in sanitary science. It is this: Conceding that certain inherent difficulties attach to the formation of communities of people, with their varied industries and geographical locations, how can the greatest possible immunity to society be secured from the evils incident thereto? It contemplates that prejudicial influences be reduced to the minimum degree of activity, while wholesome ones are given the widest scope. Latter-day observation shows, for the encouragement of the philanthropist and sanitarian, that most of the evils, of a social origin, that afflict society, are preventable; and, upon this basis, the superstructure of *preventive* medicine has been raised.

Between the individual citizen and the community of citizens there are reciprocal relations, duties and obligations. The

* See the Seventh Annual Report of Massachusetts Board of Health, 1875, page 289.

more the latter—the great body politic—can be individualized, and brought to mind as the immediate neighbor, citizen, friend, and even guest, of the former, the clearer and more practical will be the perception of the mutual responsibilities involved in this relation. If this abstract view generally obtained, it would be safe to declare that public health is the sanitary complement of individual health. Unhappily, the representative public, whom I have characterized as every man's neighbor and friend and guest, is too often recreant to duty to enjoy so great distinction. The proffered salutation of the latter is not seldom met with tainted breath, polluted drink and tarnished garments.

The purity of the "air we breathe and of the water we drink" is too often, in its keeping, lost or impaired to a degree sufficient to make the equivalent of so much poison to the unconscious consumer. Next to cleanliness, the condition of the atmosphere and the water-supply of cities constitute the most important themes of the sanitarian. It is difficult to say which should take the precedence, although it may be assumed as an axiom in science, that, in proportion to the opportunity for, and consequent degree of, contamination of these elements of healthfulness, in any given place, in a similar proportion will the foot-prints of disease be the more clearly marked. Infected air and infected water are equally hurtful. Observation proves that the water supplied to cities and towns for drinking purposes, is a frequent inlet to endemic and even epidemic diseases—cholera, typhoid fever and other epidemic disorders have been but too surely traced to the foul water furnished to portions of populous cities thus scourged.

To every city, having a specific water-supply brought perhaps from external sources, as rivers, ponds or springs, an inquiry into and constant watchfulness over its purity are by no means superfluous.

How then is Augusta supplied with water? and whence the danger threatening its purity?

Augusta is supplied with water for all purposes from three sources, namely:

1st. *Pumps* within its limits, and chiefly on the suburbs.

2d—The Turknett Spring.

3d—From the Savannah River, via the *Augusta Canal*.

I shall notice them in the order in which they are mentioned.

PUMP WATER.

There are now 118 pumps within the city limits, although situated chiefly in retired localities and on the back streets. This water was originally very pure, indeed all that could be desired; cool, palatable and pure, free from organic and inorganic impurities, it compared favorably with that subsequently supplied by the Turknett Spring. Even now new and much-used pumps in certain localities, afford wholesome water in every sense, so refreshing and cool in the summer season as to enable the population dependent upon it to dispense with ice.

These pumps vary in depth from 14 to 30 feet, the natural purity of the water being clearly foreshadowed by the geological arrangement of the soil upon which the city is built. As far back as 1860, Prof. Joseph Jones, now of New Orleans,* after a thorough investigation of the subject, remarked that "an examination of the strata upon which Augusta rests, shows that there is no cause of contamination in their chemical constitution; its soil being composed of alternate layers of sand and clay, with coarse sand and pebbles for 75 feet, and rests upon the primitive non-fosiliferous rocks."

But such were the alterations in the character of this water due to the absorption and percolation of surface water, and lime used for disinfecting purposes, this able chemist, after repeated analyses of water from pumps, then much in use, declared it the equivalent of the " drainings of graveyards," the difference being rather that of quantity than quality of the constituents. Furthermore, having specified the sources of pollution, most of which still exists to taint the atmosphere and saturate the soil, as numerous cesspools, earth-pits, open sewers with imperfect drainage, requiring the liberal annual distribution of lime in the streets and premises, not forgetting also, that then as now, the city was one vast but beautiful pasture, he adds: " we have then in the city of Augusta, the same arrangement employed by the manufacturers of nitre from manure and lime and urine," (page 293.) Since 1860, manifest improvements have been made, particularly in the substitution of covered brick drains for those unsightly central ditches that formerly

* First Report to Cotton Planters' Convention of Georgia, page 291, 1860, by J. Jones, chemist.

marred the appearance, as well as promoted the uncleanliness of the streets. It is feared, however, that, in the absence of an established system of sewerage to the completion of which all forward steps should look, the improvement is not altogether real—the contents of said drains being simply hidden or removed to other localities out of sight, it is true, but not beyond hurtful limits. With this view of it, it is no figure of speech to say, that the beautiful characters of our city do not relieve it from the imputation of being essentially, intrinsically, a mine of filth, needing on favorable occasions, the explosive spark alone to precipitate pestilence. Last fall when we stood upon the very threshhold of this disaster, a condition of filth was brought to light, frightful to contemplate, in the light of possible consequences.

Just here, allow me to say that the same causes that have contaminated the soil, and the water derived from it, have operated in other, and perhaps equally hurtful, directions. Where their effects could be made so palpable, they have led to the abandonment of the pumps at large; but, without doubt the risk has been and still is, equally great, so far as the air we breathe is concerned. Undoubtedly, the atmospheric contamination has kept pace with that of the water, and, but for the instability of the air, and the agitation to which the kindness of nature subjects it, would have, long since, been converted into a vortex of disease to engulf the people. Unhappily, there is no resource for the substitution of a more wholesome atmosphere, as in the case of water, except by devotion to that reform which looks steadfastly to the *prevention* (not removal) of *filth*, as the sole duty in the premises. This has never been done, and, I fear, never will; for, as long as our citizens are not made sensibly, and painfully, to realize the vitiation of the local atmosphere, they will remain content with an imperfect drainage and associate sources of pollution. The plea of economy and poverty is ever urged against the employment of a true sanitary reform, which only conserves, when wisely instituted, the real wealth, happiness and health of the population. Defective drainage stands at the head of the evils that afflict this, otherwise, well-ordered community, retaining, as it does, upon, or near, the surface, and that in a retentive soil, materials favorable to rapid decomposition, and promoting great humidity of the soil and atmosphere, especially in the winter and wet season, when the water level* is not far beneath.

As there are a few pumps in the interior still in use, I give the following analyses, made by Professor Jones, selecting the best and worst of them, in order that you may estimate the degree of pollution and the unwholesomeness of all present pumps similarly located. It will be observed that the analysis gives only the character and amount of mineral substances held in solution:

*Analysis* 56—*Pump-water, intersection of Broad and Washington Streets—September* 10, 1860.

100,000 grains contain:

| | |
|---|---|
| Solid residue | 43.642 |
| Nitrate lime | 17.280 |
| Chloride calcicum | 3.995 |
| Sulphate magnesia | 3.315 |
| Chloride magnesia | 8.273 |
| Chloride sodium and potassium | 6.107 |
| Nitrate sodium and potassium | 1.060 |
| Chloride ammonia | 0.397 |

*Analysis* 54—*Pump-water from intersection of Centre and Greene Streets—Sept.* 10, 1860—*Hot and dry weather.*

100,000 grains contain:

| | |
|---|---|
| Solid residue | 78.972 |
| Nitrate lime | 32.400 |
| Sulphate lime | 0.181 |
| Chloride calcium | 12.562 |
| Nitrate magnesia | 7.525 |
| Chloride magnesia | 10.749 |

If we add to this, the possible and probable organic impurities to be found, I think, Mr. President, it would be in order for this society to recommend to the Board of Health, the condemnation of all pumps ascertained by chemical examination to approximate the above.

TURKNETT SPRING WATER.

That splendid fountain, Turknett Spring, located on the south side of the Sand Hills, about two miles from the city, to the south-east, furnishes water almost as pure as crystal, and

* This sometimes approaches within twenty inches ot the surface.

nothing but the insufficiency of the supply and the great expense incident to maintaining it, could have reconciled our citizens to its withdrawal. It is almost as pure as distilled water, its specific gravity being 100.007. (Report before cited, page 206.) It contains 4.14 grs. solid residue to every gallon, or $6\frac{1}{2}$ in 100.000 parts.

It is conveyed by pine logs with iron connections to the reservoir at the upper end of Broad street, and is thence similarly distributed over the city, the sources of contamination, being within the line itself, i. e. from the destruction and decay of the logs and the oxidation of the iron joints, and also from the penetration of defective logs by offensive organic fluids. Prof. Jones says that the "reddish brown sediment which is drawn from the pipes of this system is formed by the action of the water, atmospheric air, and carbonic acid upon the iron joints of the logs."*

Since the introduction of the river water, the number of hydrants furnishing this water, has been greatly reduced, the city government placing upon them a high tax ($20.00) with a view to compelling its discontinuance by the citizens. If the river water could be subjected to complete filtration, and preserved against external sources of contamination as readily, we might cheerfully surrender the former for the more convenient and abundant supply of the latter, since, in point of chemical purity, the proportion of *mineral* matter held in solution is almost the same as will presently be seen.

This is due to the fact which Prof. Jones has pointed out, that the waters of these two streams have a common geological source, namely, the primitive regions of Georgia, and other points northward. Those then who adhere to the spring water, under the apprehension that they get a purer water than that of the river, except as to *suspended* matter (clay, etc.) deceive themselves. River water filtered for the removal of the latter is quite as pure and equally wholesome, provided its pollution from surface-water and sewage be prevented.

The City Tax Digest shows that there are now 254 hydrants subject to taxation.

* See "Report on Kaolin Pipes and Turknett Spring water," to John Davison, Esq., Member of Council and Chairman of Augusta waterworks, 1860.

RIVER WATER.

The supply from this source is obtained directly from the first level of the Augusta canal, which taps the river seven miles above the city, bringing within the reach of its industries, its sanitary and domestic requirements a volume of water equal to all prospective demands. It is a comfortable reflection to know that, while the city, since the enlargement of the canal, has at its disposal 14,000 horse-power for promoting the industrial enterprises so vital to its future growth, it also has at command the means of supplying, without stint, or detriment to them, a volume of water equal to its internal purification, even to absolute cleanliness. This cannot, perhaps, be said of any other city in this country.

In the plan of the Augusta water-works it is drawn from the first level in the vicinity of the Granite Mills, (now the Enterprise Manufacturing Company) into what is known technically as the "Receiving and Settling Basin"—thence it passes into or through the "Filtering Basin" into a "Clear Water Basin." Having thus been prepared and crudely filtered, it is conveyed by pipes to the "Pump House," near the third level of the canal, where, by means of a water and steam pump, it is forced into a reservoir, ninety feet high, and having a capacity of 160,000 gallons. Here the pressure is obtained that determines its distribution to the whole area now supplied. These pumps are capable of furnishing about 3,500,000 gallons per day, which is actually done in the summer months.

Now then, between the point of supply for the basins and that of distribution, no opportunity for the pollution of this water is afforded; not even those, bent on malicious mischief could effect its pollution, as the city provides a watchman at the basin as well for this as other obvious purposes. The purity of the supply to the city then depends upon the condition of the water drawn directly from the canal, there being no opportunity for subsequent contamination; the purity of the canal-water depends in turn upon that of the Savannah river, and of the streams that contribute to its volume between the locks and city.

Fortunately, I am able to present an analysis of the Savannah river water from the hands of the same indefatigable chemist, Prof. Joseph Jones, whose love of labor knows no bounds, and whose accuracy none will question. It gives me pleasure to refer to his labors at a time when it is hoped they are to be used for the practical benefit of our people.

*Analysis* 32—*Savannah River water opposite the city of Augusta—September* 10, 1860—*Water unusually low—The season has been hot and dry.**

Suspended matters in 100,000 grains, grains 5. The suspended matters are much less than in wet seasons, when the red clay is washed down. These suspended matters were found, under the microscope, to consist of particles of black and white mica, fine silicious sand, minute particles of different non-fossiliferous primitive rocks, animalcules and clay colored red by per-oxide of iron. One hundred thousand grains of water, after the removal of the suspened matter, contained:

| | |
|---|---|
| Solid matters | 4.2936 |
| Carbonate of lime | 0.7544 |
| Carbonate of magnesia | 0.0250 |
| Sulphate lime, chloride lime, chloride magnesia, and phosphate lime—slight traces. | |
| Chloride sodium | 0.0436 |
| Sulphate soda and potash | 0.0489 |
| Silicic acid, silicate alumnia, potash and soda, together with a small proportion of organic matter, and traces of ammonia | 3.1210 |

Making due allowance for the varying proportions of insoluble suspended matters, particularly the red clay, that immediately arrests attention in wet seasons, the Savannah River water is as pure and wholesome drinking water as can be desired, comparing very favorably with the best springs of the State. Properly filtered, it is as clear to the eye as the Turknett Spring water, although the filtration to which the authorities subject it is scarcely worthy of the name—the clay precipitate being ever present, and sometimes to such a degree as to make it a distasteful draught, which not even a julep could redeem.

Until very recently, there can be no doubt that the water of the Augusta canal was as pure at its point of delivery as at its river head; there were no proper sources of contamination, along the seven miles of its course. Even now, that which I

* Page 252 Jones' Report.

shall proceed to point out as the great source of future pollution is not a felt-injury, although active on a small scale. It is to the future evil that rises in magnitude with the lapse of time, and *pari passu* with the prosperity of the manufacturing interests of the city, and its consequent growth in population, to which attention will be called.

During the speculative era of which the present financial and commercial depression is the natural sequence, and after the recent enlargement of the canal was actually begun, a corporation, known as the " Augusta Land Company," was organized, which, with the aid of even foreign capital, purchased a large body of land west and south of the canal, between the Summerville Railroad on the south and Washington Railroad, on the north, and extending from Bohler's property, on the east, to the foot of the "*Hill*," on the west; in all, about 370 acres. On the extreme northwest, this purchase extends, I believe, quite to the mouth of Rae's Creek, where the latter amplifies into Lake Olmstead. Under the apprehension that the prospective construction of cotton and other factories (which, under the stimulus of unnatural dividends by the factories then in operation, sanguine individuals already descried in the near future) would greatly enhance its value, this company proceeded to lay the foundation for and invite the future extension of the city to this point.

Maj. J. J. Gregg, a highly public-spirited citizen, was its first President, and, with characteristic ability, he devoted himself to the preparation of this tract for future annexation. At that time much of it was a low, marshy, malarial area, so flat that the natural rainfall was often retained for days upon the surface. The first step towards utilizing it, after surveying and laying off its streets to correspond as nearly as possible with the principal streets of the city, was the adoption of a drainage system complete in its operation. Through the magnificent street, Crawford avenue, that faces the Scheutzen Platz, there runs a splendid brick underground sewer, into which the tributary sewers (surface and otherwise) empty their contents. With some exceptions, the drainage of the entire slope of the Sand Hills, on this part of its eastern exposure, finds its way into this sewer, which has its outlet, after crossing Washington road above the "Beverly Walker" residence, into the first level of the canal, whose south bank, at this and sundry other points along its course, is formed by the hillside, and not by an embankment.

Near the outlet of this sewer, a surface stream of apparently clear water from the hillside, more directly embraced in the Milledge tract, also empties; but as the latter has been surveyed, Greene street being continued to the highest point of the hill as marked by the symmetrical rows of trees, it too will sooner or later become an objectionable sewer, gladly availed of for purifying the hill-sites, but bearing polluted water into the canal.

The effect of this skillful drainage has been to recover this property from all objection on the score of unhealthfulness that heretofore attached to it. It may be truthfully said to be better drained than any portion of the city proper. Within the latter there is no system above or beneath the soil; it is all irregularity and insufficiency, while there the system only requires occupation to complete it. Its perfection is secured by providing for the continuous irrigation, if need be, of the principal sewer, by dividing the volume of water that flows from Heckle's spring, and thereabouts, one-half being turned directly into it, while the other traverses an open gravel-bed sewer, to become converted into a brick subterranean drain in the future. This is precisely what I endeavored to impress upon the Board of Health last year as a necessary practicable measure for portions of the city east and north of the third level of the canal. Continuous irrigation of the principal sewers is the beau ideal of sanitary precaution against localized filth, and, if possible, should be engrafted upon the water-carriage system of every city seeking its supply from external sources.

Now, Mr. President, if you enter the southeast corner of this property, and survey the extensive water-shed formed in part by plain and hillside, at the same time anticipating the future, so far as to behold this occupied by a population of from 5,000 to 8,000, you can then forecast the probable risk of contamination to the water of which you and I are to drink. The filth of the streets, wet waste of every kind from dwellings, sewage combining excreta of man and animals, all seeking outlet in that stream from which the city draws its supply of water for drinking and cooking.

But this is not all. I read from a pamphlet prepared by Mr. B. Holley, C. E.,* in 1875, under the direction of the Board of Managers of the Augusta Canal, as follows:

"The company also own, on the opposite side of the canal, a tract of land extending from the Washington road nearly to Rae's creek, containing ninety acres, exceedingly well located for the use of operatives. This land will be sold at very low rates."

This tract is opposite to the old "Powder Works," and forms part of the hillside, the slope being towards the canal and the sewers before spoken of, into which the sewage of the factory population, who are to find homes at this point, must be carried. The best factory sites, beyond the city limits, are found in this vicinity, and it is altogether likely that these ninety acres will be utilized for factory residences. Already a factory of 60,000 spindles is spoken of for this point, which gives seriousness to the probable injury likely to be felt in this direction. The Augusta Factory has a population, made up chiefly of the families of the operatives, of quite three thousand, so that from one factory of twice its capacity, or several aggregating this, a population of perhaps 5,000 may be expected. Adding this to the estimate for the Land Company's area, it will give a total of perhaps 10,000, the sewage of whose streets and premises must be thrown into the canal beyond possible neutralization, escape by filtration or other known means.

Assuming an increase of population equal to this in twenty years, which is a short time in the history of a city, and a generous offer to those who defend this magnificent speculation by appeals to the future, one-third of its sewage will then be incorporated in the water supply to the balance.

This is no chimera; it stands before us as a prospective reality, tangible in form and terrible in its consequences. Nothing we eat or drink could escape the pollution. Not a milk-maid could wash her pans or pails and escape the evil; the refreshing beverages of the summer would contain a due proportion of diluted sewage; the water tendered the fevered sick would contain possibly the germs of the disease from which he already suffered; all, all, of whatever class or condi-

* The Enlarged Augusta Canal, etc. By Byron Holley, C. C. 1875.

tion, must drink it, and take the consequences, whatever they be.

When this state of affairs is ushered into existence, with the progress of time, having in reality made a *sewer* of the medium of our supply, we drink nothing more nor less than sewage in different degrees of dilution. If compelled to use it, let it be "frankly recognized as unclean."*

Mr. Simon's remarks on this point are so apposite that I shall give his own language. "Regarding rivers as sources of drinking water, one of two positions ought, I submit, to be consistently aimed at; either that, being a necessary source of domestic water-supply, the river shall be absolutely protected against pollution; or else that, being (in whatever degree) used as a sewer, it shall be classed as not fit to supply drinking water."

The importance of timely action in this matter will be appreciated by the announcement that *when once contaminated with sewage to a sensible degree there is no known method of purification for safe use.*

In order that this may be set at rest, I shall cite the opinions of the most recent authorities. In the Seventh Annual Report of the Massachusetts Board of Health, for 1875, page 298, Dr. C. F. Fulsom, remarks: "No authoritative body, so far as I have been able to learn, has declared itself as fully satisfied with any other process for the purification of sewage than that of *irrigation,* that is, its application to and absorption by the soil, the action of which is not "simply mechanical;" it is chemical also, for the results of filtration, properly conducted, are the oxidation, and thereby the tranformation of the offensive organic substances, in solution in the sewage stream, into fertilizing matters which remain in the soil, and into certain harmless organic salts which pass off in the effluent water," (page 326.)

"Much indeed, he adds, has been said as to the complete self-purification of rivers by a flow of a few dozen miles. No such power exists. The solid parts are deposited, and *what remains looks clear and bright, especially when largely diluted.*

* "Filth Diseases and their Prevention," by John Simon, F.R.C.S., Chief Medical Officer of Privy Council and of Local Government Board of Great Britain, page 84.

Chemical changes take place too, sometimes decomposition, sometimes putrefaction, sometimes simple elective combinations. *If sewage contain the "germs" of disease, whatever they may be, no agency at present known, except a sufficiently high temperature, will efficiently destroy them;* "*this was the unanimous decision of the International Medical Congress at Vienna, in* 1874." "*Excessive dilution simply diminishes the chances of danger from any particular tumblerful,* (page 283.)

There is no hope then, Mr. President, either by chemical, mechanical or other means of purifying the stream whence this city derives its supply of drinking water, when the contamination foreshadowed has been brought about. To face this fact is sufficient apology for the details of this paper as well as for seeking to make it public. Let our citizens be made aware of this fact, so hurtful and so fatal to their individual welfare, and, forewarned, insist upon its *prevention*, now so easy, which is the objective point of this paper. Lest I fail to provoke that interest of alarm necessary to the latter, I will submit the following extract from Dr. Simon, on the subject of "infective water," *i. e.*, water charged with septic ferment, the prolific cause of endemic and epidemic diseases:

"In infective water, as in infective air, the zymotic (fermentated) malignity is but indirectly, and most imperfectly suggested to us by qualities which strike the common sense, or by matters which chemical analysis can specify. As any unbrutalized sense of smell will turn, with disgust, from certain airs, so will it and common taste and sight, be repelled by certain waters; and, as the chemist can show certain foulnesses in the one, so he can show certain foulnesses in the other; but these tests, it must always be remembered, are tests, only, of the most general kind. Confessedly they do not touch the *corpus delicti*, but only certain conditions to which it is, or may be, collateral; and their negative findings are, consequently, not entitled to the same sort of confidence as their positive. Chemical demonstration of unstable nitrogenous compounds in water is a warning which, of course, should never be disregarded; but, till chemistry shall have learned to identify the morbific ferments themselves, its competence to declare them absent, in any given case, must indirectly be judged incomplete, and water which chemical analysis could, probably, not condemn, may certainly be carrying in them very fatal seeds of infection." (Pages 19 and 20.)

These ferments, or septic germs of disease, are literally unknown, except by their consequences. Cholera, typhoid fever, and other diseases of like character, have a portable poison, or germ, which, according to professional opinion of the present day, is taken, most likely, with infected water; these, and other sources of danger, are beyond the eye of the microscopist, the reach of the chemist, or other tangible method of separation and identity. They belong to the sphere of the unseen, (but not unfelt) parasitic world around and about us, which is as yet an impenetrable mystery, just enough being dimly recognized of its outlines to confuse. That a way of successful approach, in the further progress of knowledge, will some day be opened up, I doubt not. Meanwhile, so far as the water we drink is concerned, our only safety is found in preventing consequences, as far as may be, by *excluding all possible sources of contamination.*

By way of enforcing much that has been said, even at the risk of repetition, I will submit the following extracts from the Seventh Annual Report of the Massachusetts Board of Health.

"The opinion of the English Rivers Pollution Commission was very decided on the subject of the use of polluted water," (1868.) "No process has-yet been devised of cleansing surface-water and water contaminated with sewage, so as to make it safe for drinking." And, again, "among the numerous processes for the cleansing of polluted water, there is not one sufficiently effective to warrant the use for drinking of water which has once been contaminated with sewage or other similar noxious animal matters."

"If this view of the case may seem to be over-cautious, it is to be remembered that the poison, however trifling, is taken daily, and that although when in robust health, the individual will not suffer from it, it may be sufficient to make itself felt when he is prostrated by sickness and his powers of resistance to such influence are then proportionally impaired," (page 44.)

Again, "a water supply, whose quality is *somewhat* deteriorated by the constant admixture of sewage, but not to such a degree as to lead the chemist to *condemn* it, or to cause distinct disease or increased death-rate among its consumers, may yet gradually and insiduously lower their vigor, so that when the time comes, as come it must in the case of streams which receive sewage from privies and water-closets, that some of the above mentioned evacuations (cholera and typhoid fever) enter a water-supply, the zymotic poison than conveyed to the damaged constitutions of those using it, will find them prepared to yield to the morbific influence, as the feeble and sickly everywhere succumb to disease which stronger and healthier constitutions resist successfully," (page 118.)

Again, in a report of a Medical Commission, composed of Drs. Swan, Wood and Bowditch, to the city of Boston, "upon the sanitary qualities of the Sudbery, Mystic Shawshene and Charles river waters," in 1874, the following memorable reference to this subject is made. "The water-supply should be uncontaminable by drainage. There is no demonstrable safety in a middle course. No one has conclusively shown that it is safe to trust to dilution, storage, agitation, filtration or period of time for the complete removal of disease-producing elements, whatever they may be. Chemistry and microscopy cannot and do not claim to prove the absence of these elements in any specimen of drinking water. They discover pollution; and pollution is somehow intimately associated with the production of certain diseases. Our germ, fermentation and other theories indicate our actual ignorance of the ultimate nature of that cause or those causes whose action we nevertheless rightly infer. These deductions from the observation of cases and their surroundings have been so often repeated that we cannot afford to disregard them. Chemistry in these cases has been invaluable in pointing out the fact and source of contamination but it has not indicated the quantity and quality of the specific cause," (page 231.)

The enormity of the offense against decency and the cleanly instincts of human nature, to say nothing of sanitary science, by the permission of the forthcoming evil, may be forcibly illustrated by figures—a very tangible as well as useful method to those who may be disposed to regard its presentation as a future problem as over-strained and perhaps out of season. A population of 10,000, such as we have pre-figured, according to the most available physiological data within my reach, would yield annually quite 500 tons of manure, which, if promptly discharged into the canal, would give nearly 3,500 pounds daily. If we add to this the night-soil of the streets, household and kitchen slops, everything indeed that can be included in the "wet-waste" of households, then the curtain is raised upon a contingent of filth shocking to contemplate, much less to risk.

Such, Mr. President, is the future outlook to one who would guide the footsteps of his fellow-citizens by the teachings of science, by enlightened experience, as well as by the instincts that prompt to self-preservation. Happily, the diversion of the sewage under comment is an easy matter at present, and involves no injury to important interests. The Augusta Canal is the opening bud of promise for this city, and even now the harvest of the future is within sight: the key to its increase of population and growth in commercial importance as a manufacturing centre, let it not become the inlet to that worst enemy of municipal progress, endemic and epidemic sickness. The reputed healthfulness of Augusta is a part of the capital employed to attract those given to manufacturing pursuits. "Health is wealth" in an important sense, both to capital and labor. In the effort to attract and secure the latter to build our waste places and restore the lost fortunes of an impoverished people, let us retain that part of the means necessary to our recuperation that a beneficent Providence has vouchsafed us, namely, the assured conditions of health as embraced in the supply of pure water—pure as it falls from the heavens and issues from the everlasting rocks.

In conclusion, Mr. President, I offer the following resolution.

*Resolved,* That the Augusta Library and Medical Society respectfully call the attention of the City Council to the circumstances threatening the water supply of the city, and recommend such legislation on their part as will effectually prevent its further or future contamination.

